# Ponticulus posticus and skeletal malocclusion: A pilot study in a Southern Italian pre-orthodontic court

**DOI:** 10.1515/med-2024-0965

**Published:** 2024-07-15

**Authors:** Claudia Lombardo, Federico Roggio, Rosario Caltabiano, Grazia Maugeri, Grazia Agata D’Amico, Antonino Lo Giudice, Giuseppe Musumeci, Carla Loreto

**Affiliations:** Department of Biomedical and Biotechnological Sciences, Section of Anatomy, Histology and Movement Science, School of Medicine, University of Catania, Catania, Italy; Research Center on Motor Activities (CRAM), University of Catania, Catania, Italy; Department of Medical and Surgical Sciences and Advanced Technologies “G.F. Ingrassia” Anatomic Pathology, University of Catania, Catania, Italy; Department of Drug and Health Sciences, Section of System Biology, University of Catania, Catania, Italy; Department of General Surgery and Surgical-Medical Specialties, School of Dentistry, University of Catania, AOU “Policlinico-San Marco”, Catania, Italy

**Keywords:** ponticulus posticus, atlas, teleradiography, bony protusion, malocclusion, vertebral anatomical variant

## Abstract

**Purpose:**

Ponticulus posticus (PP) is a bony protrusion located between the posterior portion of the superior articular process and the posterolateral portion of a posterior arch of the atlas vertebrae in the cervical spine. The aim of this study is to verify the presence of different types of PP in a Southern Italian pre-orthodontic cohort to understand its correlation with skeletal class and maturity.

**Methods:**

A case–control retrospective study was conducted, utilizing 212 latero-lateral telecranium radiographs to analyze skeletal maturity according to the cervical vertebral maturation method, the Angle’s classification of malocclusion (I, II, or III), and the presence or absence of the PP, whether complete (c-PP) or partial (p-PP). A total of 212 lateral cephalograms were analyzed.

**Results:**

Of the 72 male patients, 67 (93%) exhibited PP, and 116 (88%) were PP. The chi-square value was 0.001, while Cramer’s *V* was 0.270, indicating a significant correlation between age groups and PP presence, and a very strong association overall. Out of the 41 complete PP cases, class I was notably more prevalent than classes II and III.

**Conclusion:**

Orthodontists should carefully consider PP when assessing and treating individuals with or without skeletal discrepancies and dental anomalies.

## 1 Introduction

The ponticulus posticus (PP) is a bony protrusion situated between the posterior portion of the superior articular process and the posterolateral portion of the superior margin of the posterior arch of the atlas vertebrae in the cervical spine. It is also known as Kimmerle’s anomaly or the arcuate foramen [1,2] and represents one type of craniovertebral junction anomaly (CVJ). CVJs encompass various structural abnormalities at the interface between the skull and cervical spine, potentially leading to neurological complications due to compression of vital structures. Menezes divided CVJs into “congenital” (e.g., basilar invagination, atlas assimilation) and “developmental (acquired)” types (e.g., acquired BI, rotary dislocation, os odontoideum, syndromic conditions), as shown in Table 1 [3,4]. These anomalies can cause diverse neurological issues due to the compression of vital neural structures. The PP, an osseous bridge between the atlas (C1) and axis (C2) vertebra, is one such CVJ with the potential to impact neurovascular structures. Other notable examples include atlantoaxial subluxation (misalignment of the atlas and axis), basilar invagination (upward migration of the odontoid process into the foramen magnum), and occipitalization of the atlas (fusion of the occipital bone and atlas). These anomalies necessitate careful management to address their complex pathophysiology and associated risks.

**Table 1:** Classification of the anomalies of the craniocervical junction according to Menezes

**Congenital**
Proatlas segmentation failure
Basilar invagination
Atlas assimilation
Condylar hypoplasia
Absent components of the atlas
Spondylolysis C2–C7
Hemivertebrae, segmentation failures
**Developmental**
Basilar invagination
Rotary dislocation
Os odontoideum
Osteogenesis imperfecta
Syndromic abnormalities
Skeletal dysplasias
Goldenhar syndrome
Spondyloepiphyseal dysplasia
Conradi

The PP is an anatomical variation observed in about 15–20% of the population and, although generally asymptomatic, it has been associated with various clinical conditions such as headache, neck pain, diplopia, dysarthria, dysphagia, and vertigo, due to vascular flow reduction following vertebral artery compression [5–8]. The embryological origin of PP is not clear, and several hypotheses exist. One hypothesis suggests that it might originate from the dorsal arch of the proatlas, as suggested by the presence of lamellar patterns within the bone matrix and the cortex indicating endochondral ossification. Another theory suggests that the PP may protect the passage of the vertebral artery during cranial and neck movements [9,10].

**Figure 1:**
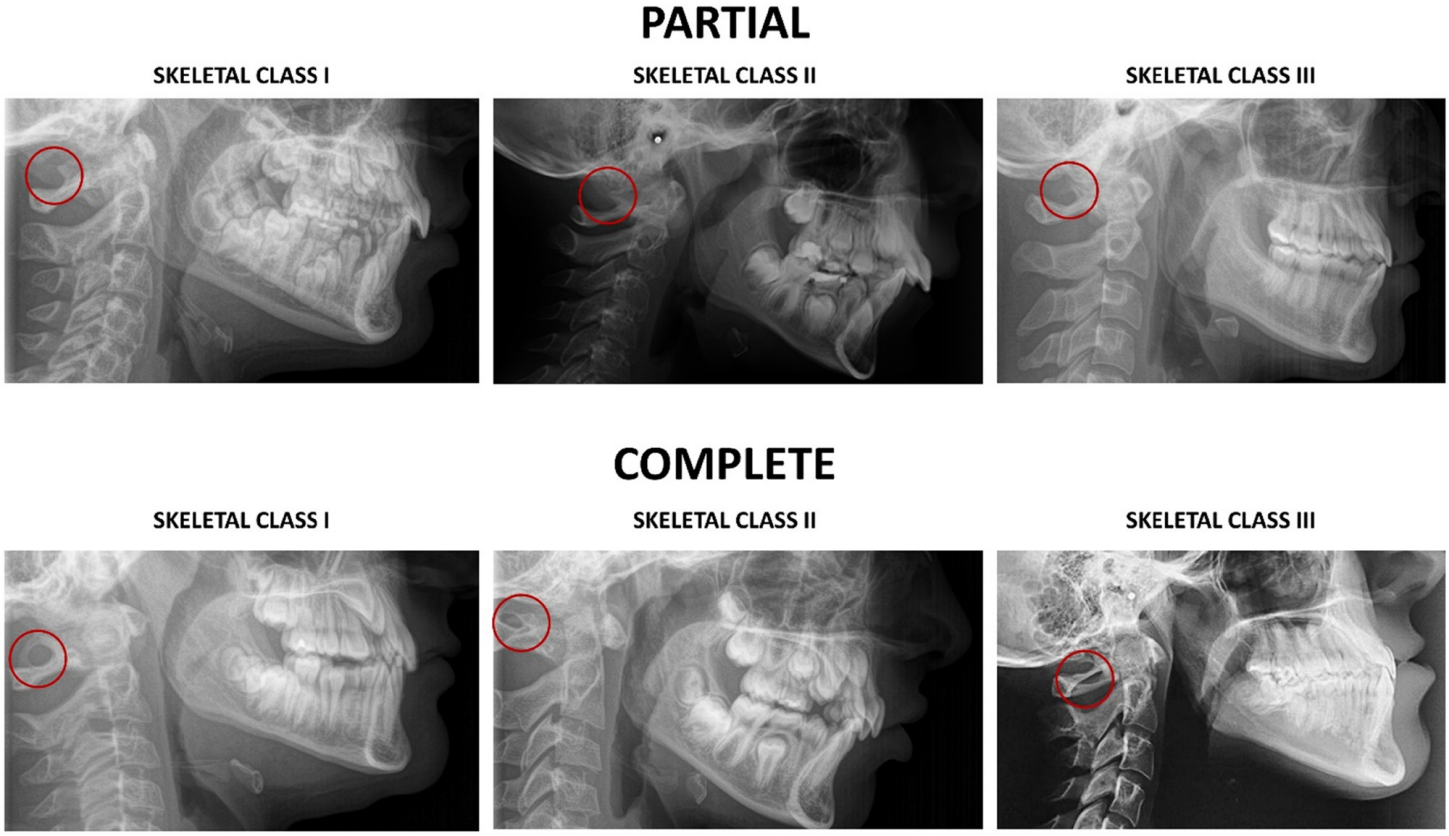
Radiographic representation of the complete or incomplete PP presence divided by the Angle’s classification of malocclusion (I, II, or III).

PP can be diagnosed through latero-lateral telecranium radiographs [11] commonly used for the orthodontic evaluation or through cone beam computed tomography (CBCT) (Figure 1) [12]. The relationship between the presence of PP and age, gender, Angle’s skeletal classes, and skeletal mandibular growth remains controversial. Some studies found PP more prevalent among males, particularly in those with a class III malocclusion [13,14]. However, other studies found no differences in the presence of PP based on gender, skeletal maturity stages, or skeletal malocclusion from radiographs of participants in the United States and Canada [15]. Despite extensive investigation, the presence of PP and its impact on various aspects of anatomy and health continue to be a topic of discussion. Specifically, the presence of PP cannot be generalized across all ethnicities, as results from various authors are often geared towards specific parameters. For example, recent studies specify the ethnicities involved in the study because the presence of PP appears to have an ethnicity background, as found among Malaysian [16], Indian [1], Korean [5], and Chinese [10] populations.

From an orthodontic perspective, it is essential to evaluate the presence of PP because it may affect the mandible’s growth pattern and thus impact orthodontic treatment planning. It is particularly important to consider the presence of PP when evaluating and treating individuals with skeletal discrepancies, both for class II and class III individuals [17,18]. In fact, different studies have associated the presence of PP with dental anomalies, particularly in the mandibular molar region. A study by Kaya et al. [19] reported a higher incidence of mandibular molar hypoplasia in individuals with PP compared to those without. Similarly, a study by Putrino et al. [20] found a significant association between the PP and dental agenesis of mandibular second molars. In addition to hypoplasia and agenesis, displacement of mandibular molars has been associated with the PP. Other authors [21–23] reported a higher prevalence of tooth impaction in individuals with sella turcica bridging, PP calcification, and other dental anomalies compared to those without. In addition, the presence of PP has been linked to other conditions that can affect overall health, such as headache and cervical pain syndrome. Numerous studies have reported associations of PP with headache and cervical pain syndrome, migraine, onset of hearing loss, and chronic tension-type headaches [24–26].

Few studies have examined the correlation between PP and skeletal malocclusion in the Italian population without yielding significant results [10,27]. As mentioned earlier, the ethnicity factor may play a key role in the association between PP, skeletal class, or skeletal maturity. Therefore, the aim of this study is to verify the presence of different types of PP in a Southern Italian pre-orthodontic cohort to understand its correlation with skeletal class and maturity. This understanding would enhance clinical and therapeutic evaluation of skeletal disorders and malocclusion.

## 2 Materials and methods

### 2.1 Patients

A case–control retrospective study was conducted, utilizing 212 latero-lateral telecranium radiographs to analyze skeletal maturity according to the cervical vertebral maturation (CVM) method (PMID: 12169031), the Angle’s classification of malocclusion (I, II, or III), and the presence of the PP, whether complete (c-PP) or partial (p-PP). The study was carried out in accordance with the ethical board of the Policlinic University Hospital of Catania “G. Rodolico-San Marco” under the title “Quantitative and Qualitative Morphological Analyses of Specific Maxillary and Mandibular Anatomical Regions (A.Q.A.M. 119/2020/PO)” and was conducted in accordance with the Declaration of Helsinki. All participants provided their informed consent prior to participating. The cephalometric radiographs were acquired by Orthophos S 3D 8 × 8 Dentsply Sirona at the following settings: 200–240 V, 50/60 Hz, max 12 A, 0.9 s exposure. The participant age range spanned from 7 to 24 years old, comprising 132 females and 80 males. The inclusion criteria included high-quality lateral cephalometric and panoramic radiographs with clear visualization of the first cervical vertebrae and being of Caucasian race. Conversely, those with craniofacial anomalies or syndromes, prior traumas, previous orthodontic or surgical treatments, poor X-ray quality, significant jaw deviation, or those of other ethnicities were excluded.

### 2.2 Radiographic evaluation

Two independent observers (CL, ALG), with decades of experience in oral and craniofacial radiology, analyzed the cephalograms to identify PP presence and its morphology. A total of 212 lateral cephalograms were analyzed. Eight radiographs were excluded due to incomplete visualization of the cervical vertebrae or poor image quality, and 21 radiographs were excluded due to the absence of the PP. To avoid evaluation errors due to fatigue, it was decided to examine 20 latero-lateral radiographs at a time. In cases of disagreement between the two examiners, a third expert in cranial radiology was consulted. Two months after the initial examinations, 100 radiographs were randomly selected and re-examined by one radiologist and one orthodontist. In instances of disagreement, a third observer was brought in. If consensus could not be achieved, the radiograph was withdrawn from further analysis.

### 2.3 Statistical analysis

All data were collected in a Microsoft Excel spreadsheet and subsequently subjected to statistical analysis using R Project for Statistical Computing (Vienna, Austria). We initially reported the data using descriptive statistics. Then, statistical differences between groups were tested using Pearson’s chi-square test for association between the variables and Cramér’s *V* to measure the strength of association. We analyzed the association between the presence of PP with, skeletal class of occlusion, age ranges, and CVM.


**Informed consent:** Informed consent was obtained from all subjects involved in the study.
**Ethical approval:** The study was carried out in accordance with the ethical board of the Policlinic University Hospital of Catania “G. Rodolico-San Marco” under the title “Quantitative and Qualitative Morphological Analyses of Specific Maxillary and Mandibular Anatomical Regions” (A.Q.A.M. 119/2020/PO).

## 3 Results

The sample examined included 72 males and 132 females, with an average age of 11.8 ± 3.94. Table 2 shows the sample distribution by gender, detailing the number with partial or c-PP. Of the 72 male patients, 67 (93%) exhibited PP, with 54 (29%) being p-PP and 13 (7%) c-PP. Among the 132 female patients, 116 (88%) exhibited PP, with 88 (48%) being p-PP and 28 (15%) c-PP. The chi-square value was 0.578, indicating no statistically significant difference in PP occurrence between males and females.

**Table 2:** Study samples that present PP either complete or incomplete

Skeletal class	Patients	Patients with PP	Partial, *n* (%)	Complete, *n* (%)	Chi-square
Males	72	67	54 (29%)	13 (7%)	0.578
Females	132	116	88 (48%)	28 (15%)	

Data were analyzed based on three divisions: presence of p-PP and c-PP stratified by age range, skeletal class, and CVM.

**Figure 2:**
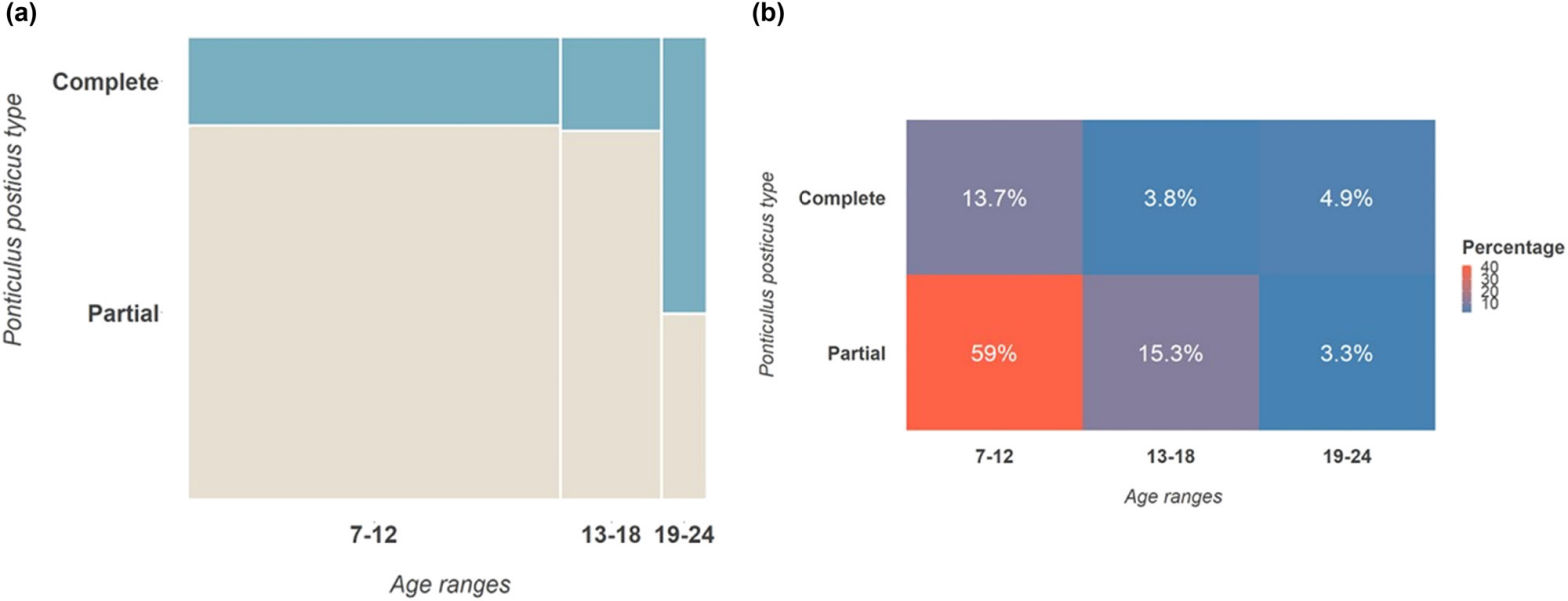
(a) Mosaic plot between age ranges and PP type. (b) Heatmap of the strength of association between age ranges and PP type.

**Figure 3:**
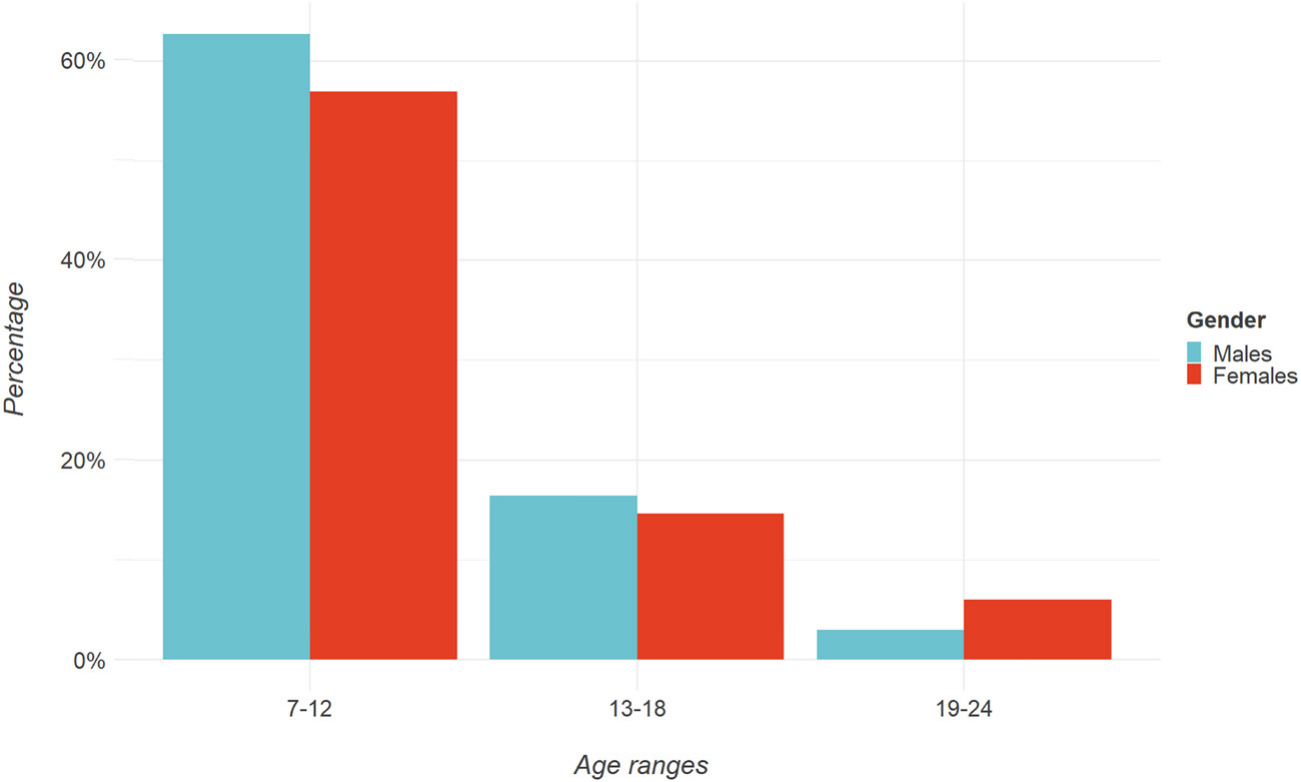
Barplot representation of gender percentages for age ranges.

Concerning age ranges, the cohort was divided into three categories, as shown in Table 3, Figures 2 and 3. Most patients in the 7–12 years group had p-PP (108, 59%), whereas 25 (14%) had c-PP. In the 13–18 years group, 28 patients (15%) had p-PP and 7 (4%) had c-PP. In the 19–24 years group, 6 (3%) had p-PP, and 9 (5%) had c-PP. The chi-square value was 0.001, while Cramer’s *V* was 0.270, indicating a significant correlation between age groups and PP presence, and a very strong association overall.

**Table 3:** Age ranges divided for partial and complete PP

Age ranges	Partial, *n* (%)	Complete, *n* (%)	Chi-square	Cramer’s *V*
7–12 years	108 (59%)	25 (14%)	0.001	0.270
13–18 years	28 (15%)	7 (4%)		
19–24 years	6 (3%)	9 (5%)		

**Figure 4:**
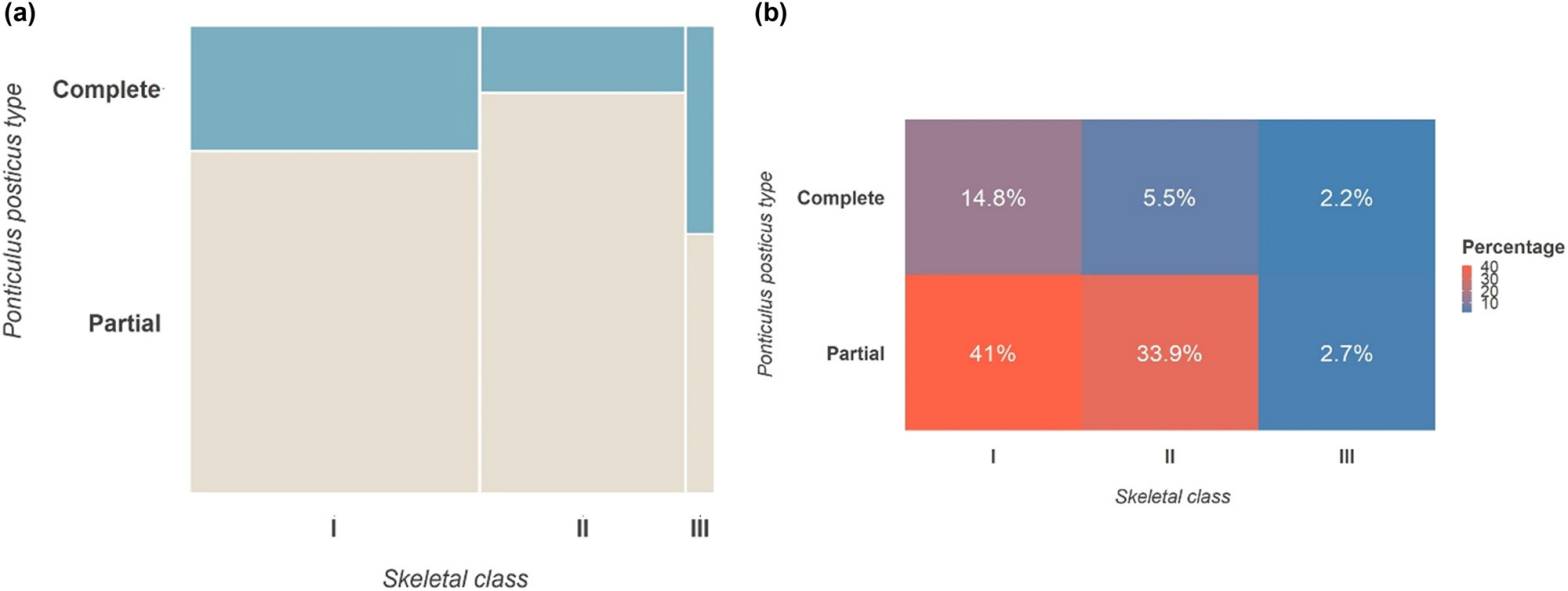
(a) Mosaic plot between skeletal class and PP type. (b) Heatmap of the strength of association between skeletal class and PP type.

**Figure 5:**
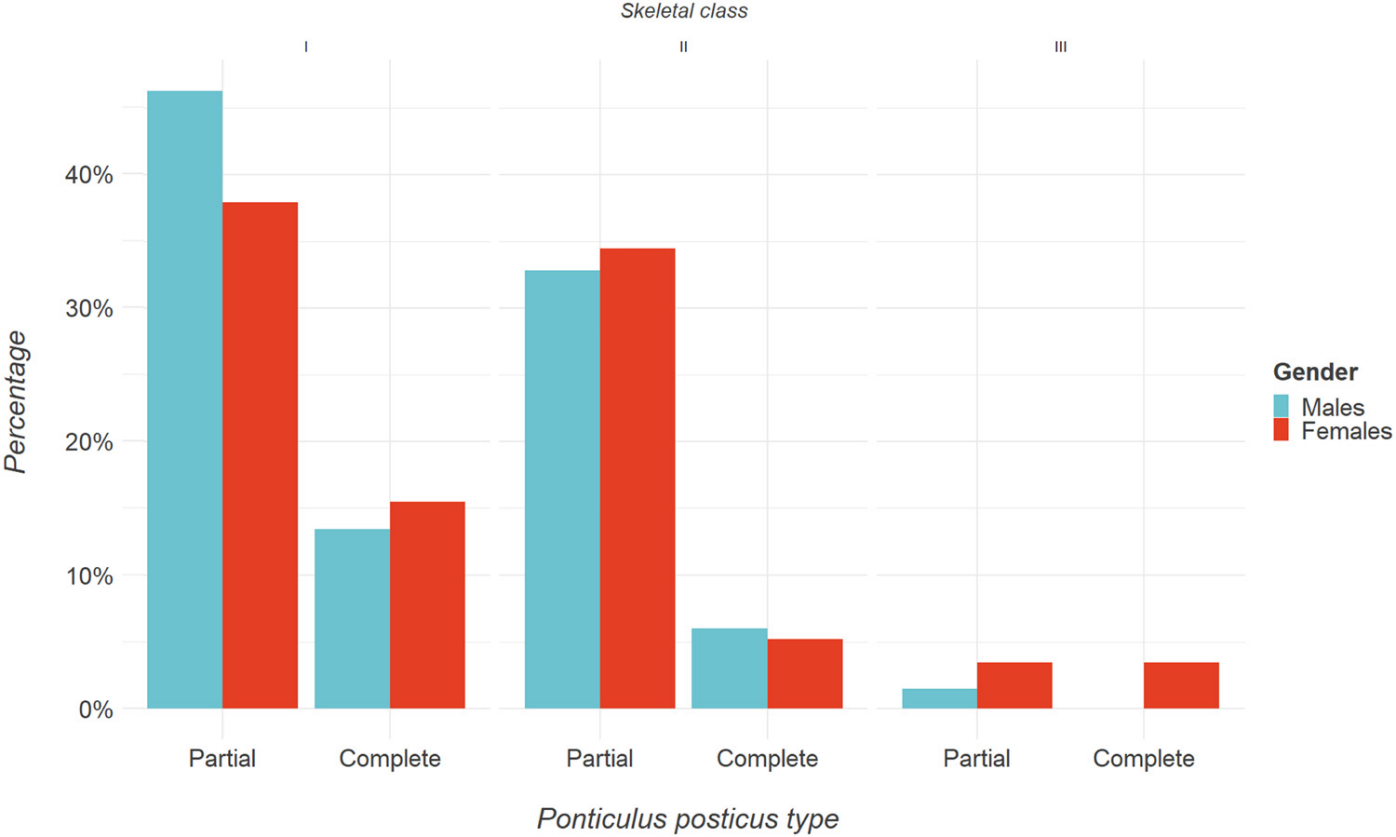
Bar plot representation of gender percentages for skeletal class.

Regarding skeletal class, we categorized patients into classes I, II, and III, as in Table 4, Figures 4 and 5. In class I, 75 patients (41%) had p-PP and 27 (15%) had c-PP. For class II, 61 patients (34%) had p-PP and 10 (5%) had c-PP. For class III, 5 patients (3%) had p-PP and 4 (2%) had c-PP. The chi-square was 0.043 for all skeletal classes, while Cramer’s *V* was 0.188, suggesting a significant association between skeletal class and PP presence, and a strong association overall.

**Table 4:** PP presence classified for the skeletal class levels

Skeletal class	Partial, *n* (%)	Complete, *n* (%)	Chi-square	Cramer’s *V*
Class I	75 (41%)	27 (15%)	0.039	0.188
Class II	62 (34%)	10 (5%)		
Class III	5 (3%)	4 (2%)		

**Figure 6:**
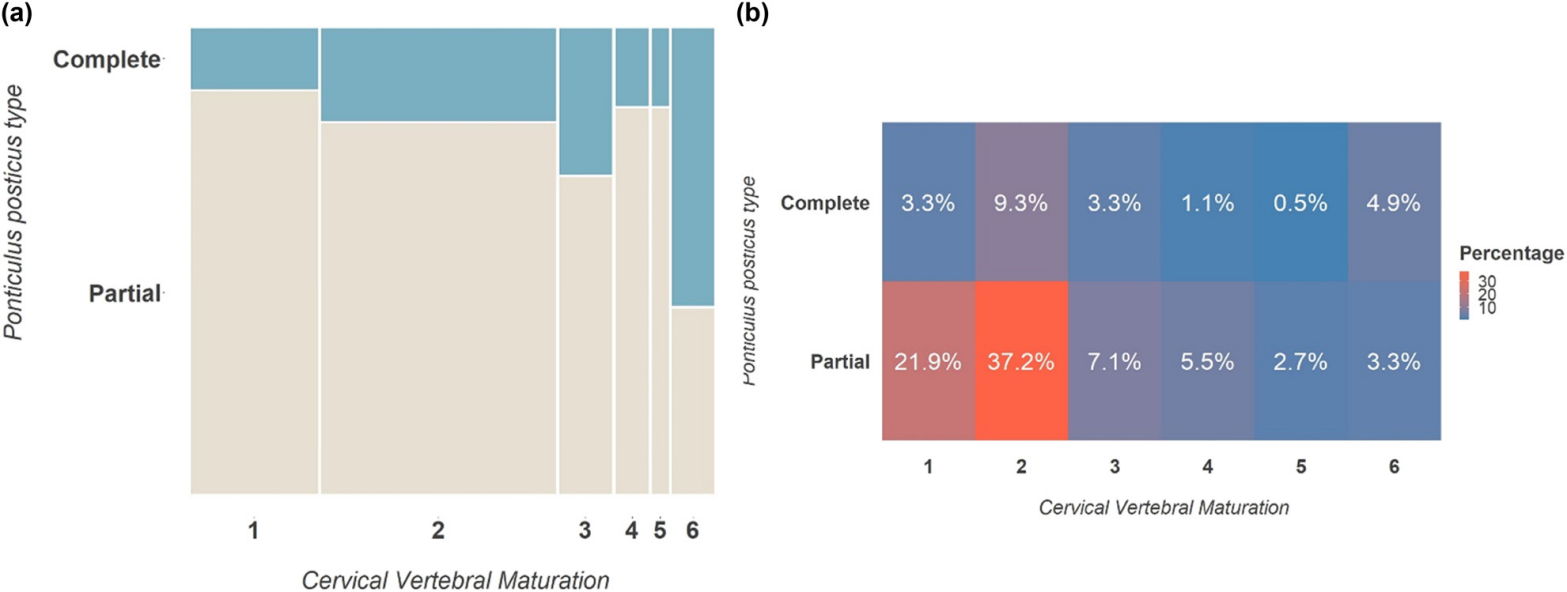
(a) Mosaic plot between CVM and PP type. (b) Heatmap of the strength of association between CVM and PP type.

**Figure 7:**
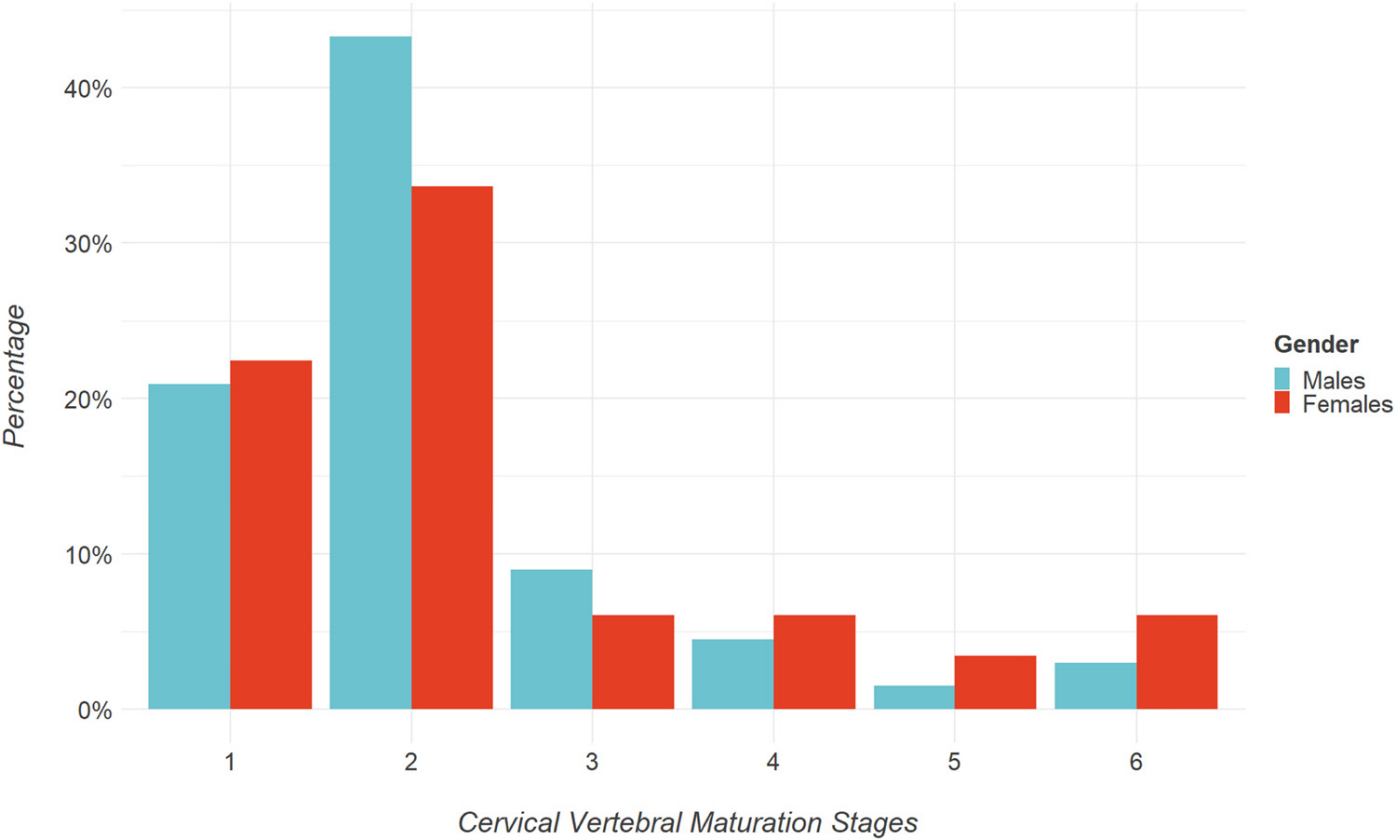
Barplot representation of gender percentages for skeletal class.

For CVM, we used the stage classification ranging from 1 to 6. Initially, we categorized the maturation stage by age range, observing a majority presence of stages 1–2 in the 7–12 age range (70%), the prevalence of stages 3–5 in the 13–18 age range (17%), and stage 6 in the 19–24 age range (8%), as reported in Table 5, Figures 6 and 7. We then examined the prevalence of p-PP and c-PP based on the different stages of CVM, Table 6. The chi-square value was 0.007, while Cramer’s *V* was 0.300, suggesting a significant relationship between CVM and PP presentation, and a very strong association overall.

**Table 5:** CVM classified for age ranges

CVM	7–12 years	13–18 years	19–24 years	Chi-square
Stage 1	46 (25%)	0 (0%)	0 (0%)	<0.001
Stage 2	83 (45%)	2 (1%)	0 (0%)	
Stage 3	2 (1%)	17 (9%)	0 (0%)	
Stage 4	2 (1%)	10 (5%)	0 (0%)	
Stage 5	0 (0%)	6 (3%)	0 (0%)	
Stage 6	0 (0%)	0 (0%)	15 (8%)	

**Table 6:** PP presence classified for the CVM

CVM	Partial, *n* (%)	Complete, *n* (%)	Chi-square	Cramer’s *V*
Stage 1	40 (22%)	6 (3%)	0.007	0.300
Stage 2	68 (37%)	17 (9%)		
Stage 3	13 (7%)	6 (3%)		
Stage 4	10 (6%)	2 (1%)		
Stage 5	5 (3%)	1 (1%)		
Stage 6	6 (3%)	9 (5%)		

## 4 Discussion

The incidence of PP in the Southern Italian population has not yet been investigated enough. This study aimed to analyze the lateral cephalograms of 212 adolescents to investigate the prevalence of the PP in relation to skeletal class malocclusion, gender, chronological, and skeletal age. The current study also found that PP prevalence was higher in females than males, although the difference was not statistically significant. We observed that the presence of PP was significantly more common in individuals with skeletal Class I malocclusion when compared to those with class II and class III malocclusions. A deeper analysis of the age distribution revealed that PP (both in its complete and incomplete forms) was most prevalent among patients aged 7–12 years. To further understand this trend, we used the CVMS to assess the skeletal maturation of patients exhibiting [28]. Our analysis indicated that PP was most frequently observed in stage 2 of CVMS. Interestingly, this stage aligns closely with the aforementioned age group of 7–12 years. While various theories attempt to explain the origin of this vertebral anomaly [10], our findings lend support to the idea that PP might have genetic or congenital roots, as proposed by Lambert and Zivanović [6]. Based on our data, it seems that the onset of PP is predictable, often manifesting during early childhood and puberty, specifically between ages 7 and 12. In contrast, its occurrence becomes rare post-puberty. For the lateral cephalograms with complete PP, the distribution of skeletal malocclusions was as follows: class I at 15%, class II at 5%, and class III at 2%. Out of the 41 complete PP cases, class I malocclusion was notably more prevalent than class II. In lateral cephalograms with incomplete PP, the malocclusion distribution was: Class I at 41%, class II at 34%, and class III at 3%. Among these 142 incomplete PP cases, class I malocclusion again emerged as significantly more common than class II. Furthermore, in lateral cephalograms with a normal atlas, all 21 cases were exclusively categorized under skeletal class I malocclusion.

The findings of this study differ from those of other studies, with some showing that the frequency of PP is higher in class II individuals and the presence of PP may affect craniofacial morphology, especially in class II individuals, leading to a shorter maxilla and a more obtuse mandibular plane angle [21]. Other authors found a significant correlation between PP and mandibular retrognathism in class II individuals [19,22,29,30] They found a significant correlation between the presence of PP and mandibular retrognathism, suggesting that individuals with class II skeletal patterns and a PP are more likely to have a retrognathic mandible. These different results probably could be attributed to the differences in the geographic areas and patient ethnicities but these divergent data could also be attributed to the different methods of identification of this anatomical variant. Several authors have found PP through CBCT, computed tomography images [9,12], or directly during cadaver dissections [6,7]. Certainly, 3D diagnostic investigations allow a particularly accurate diagnosis of PP [5,31,32], despite that this study was conducted on a series of patients who had undergone latero-lateral telecranium radiographs, as a routine diagnostic test following orthodontic treatment. Concurring with our findings, other researchers have also shown that the prevalence of PP is significantly higher in patients with skeletal class I malocclusion compared to those with skeletal class II and class III malocclusions. Moreover, these studies found a higher prevalence of PP in females than in males [14,29,30].

In the present study, we suggest that PP represents an anatomical variation that has been associated with various clinical conditions and may have implications for orthodontic treatment planning. Its correlation with Angle’s skeletal classes, particularly class I skeletal patterns, suggests that clinicians should consider the presence of PP when evaluating and treating individuals with or without skeletal discrepancies and dental anomalies. The limitations of the present study may be related firstly to the bidimensional latero-lateral telecranium radiographs which, compared with three-dimensional radiographs, may not detect the presence of PP [9,13], furthermore to the chronological age of the patients since the PP calcification process could complete at an older age and at the end of skeletal growth [7,33].

In conclusion, our study has highlighted that PP is a common anatomical variation in the Southern Italian population, often associated with dental and skeletal malocclusion. We observed a higher incidence of PP in Angle class I patients, statistically significant compared to classes II and III. The clinical relevance of this anatomical variant suggests that orthodontists should carefully consider the PP presence when assessing and treating patients with or without skeletal discrepancies and dental anomalies. Further studies are needed to deepen our understanding of the mechanisms of association between PP and different skeletal biotypes. Specifically, exploring how physiotherapic interventions may influence the management of this anatomical variant could provide valuable insights. This could contribute to the development of more personalized treatment protocols, aiming to optimize clinical outcomes for patients with PP.
